# Response Surface Modeling and Optimization of Enzymolysis Parameters for the In Vitro Antidiabetic Activities of Peanut Protein Hydrolysates Prepared Using Two Proteases

**DOI:** 10.3390/foods11203303

**Published:** 2022-10-21

**Authors:** Wedad Q. AL-Bukhaiti, Sam Al-Dalali, Anwar Noman, Silin Qiu, Sherif M. Abed, Sheng-Xiang Qiu

**Affiliations:** 1 Program of Natural Product Medicinal Chemistry, Key Laboratory of Plant Resources Conservation and Sustainable Utilization, Plant Research Center, South China Botanical Garden, Chinese Academy of Sciences, Guangzhou 510650, China; 2 South China National Botanical Garden, Guangzhou 510650, China; 3 University of Chinese Academy of Sciences, Beijing 100049, China; 4 Department of Food Science and Technology, Faculty of Agriculture and Food Science, Ibb University, Ibb 70270, Yemen; 5 Department of Agricultural Engineering, Faculty of Agriculture, Foods and Environment, Sana’a University, Sana’a 13060, Yemen; 6 Food and Dairy Science and Technology Department, Faculty of Environmental Agricultural Science, Arish University, North Sinai 45511, Egypt

**Keywords:** hydrolysis optimization, peanut hydrolysates, antidiabetic activity, *α*-amylase, *α*-glucosidase

## Abstract

Optimization of the enzymolysis process for preparing peanut protein hydrolysates using alcalase and trypsin was performed by employing the central composite design (CCD) of response surface methodology (RSM). The independent variables were solid-to-liquid ratio (S/L), enzyme-to-substrate ratio (E/S), pH, and reaction temperature, while the response variables were degree of hydrolysate (DH), *α*-amylase, and *α*-glucosidase inhibitory activity. The highest DH (22.84% and 14.63%), *α*-amylase inhibition (56.78% and 40.80%), and *α*-glucosidase inhibition (86.37% and 86.51%) were obtained under optimal conditions, which were S/L of 1:26.22 and 1:30 *w*/*v*, E/S of 6% and 5.67%, pH of 8.41 and 8.56, and temperature of 56.18 °C and 58.75 °C at 3 h using alcalase (AH) and trypsin (TH), respectively. Molecular weight distributions of peanut protein hydrolysates were characterized by SDS-PAGE, which were mostly ˂10 kDa for both hydrolysates. Lyophilized AH and TH had IC_50_ values of 6.77 and 5.86 mg/mL for *α*-amylase inhibitory activity, and 6.28 and 5.64 mg/mL for *α*-glucosidase inhibitory activity. The IC_50_ of AH and TH against DPPH radical was achieved at 4.10 and 3.20 mg/mL and against ABTS radical at 2.71 and 2.32 mg/mL, respectively. The obtained hydrolysates with antidiabetic activity could be utilized as natural alternatives to synthetic antidiabetics, particularly in food and pharmaceutical products.

## 1. Introduction

Diabetes mellitus (DM) is rapidly increasing and is expected to reach epidemic proportions around the world, particularly in developing countries [1]. Diabetes is a common chronic metabolic disease characterized by abnormal blood glucose levels caused by the pancreas’ inability or inefficiency to produce insulin, as well as changes in lipid, carbohydrate, and protein metabolism [2]. It is classified into two types: Type 1 insulin-dependent DM (T1DM), which accounts for 10%, and type 2 non-insulin-dependent DM (T2DM), which accounts for 90% of all DM cases [3]. Sedentary lifestyle, unhealthy diet, ageing, increased urbanization, and an increase in the body-mass index are all linked to an increase in the prevalence of diabetes [4].

The hyperglycemic state disrupts cellular homeostasis and biochemicals, a hallmark of diabetes [5]. These disturbances also lead to profound apoptosis of *β*-cells and inflammation-related insulitis, leading to a decrease in *β*-cells mass and function [6]. In addition to inducing non-enzymatic glycosylation of many macromolecules and the generation of reactive oxygen species, it alters endogenous antioxidants, leading to chronic complications in diabetes [2]. The regulation of postprandial hyperglycemia is associated with specific enzymes such as *α*-amylase, *α*-glucosidase, and dipeptidyl peptidase IV (DPP IV), which have been identified as therapeutic targets [3]. During the digestion process, *α*-amylase breaks down complex polysaccharides into oligosaccharides, which are then broken down further by *α*-glucosidase to release glucose, which is absorbed in the intestines and enters the bloodstream. Therefore, inhibition of these enzymes may assist in reducing postprandial hyperglycemia by inhibiting carbohydrate enzymolysis, which may delay glucose absorption in the small intestines [2]. 

Some foods contain several bioactive compounds that may benefit T2DM patients, including dietary protein and its derived peptides [7]. To manage T2DM, a change in lifestyles, such as eating habits or medication, is required; however, some medications are expensive and may cause toxicity and other negative side effects, such as kidney injury risks due to the use of glucagon-like peptide-1 (GLP-1) receptor agonists [8]. Acarbose, voglibose, and miglitol are widely used as inhibitors of *α*-amylase and *α*-glucosidase in T2DM patients [2]. Furthermore, some diabetes medications include dipeptidyl peptidase IV (DPP IV) inhibitors, sulfonylureas, biguanides, sodium-glucose co-transporter-2 (SGLT2) inhibitors, and peroxisome proliferator-activated receptor-*γ* (PPAR*γ*) agonists. Nevertheless, long-term use of these medications and inhibitors may cause serious side effects such as hypoglycemia, digestive system problems, possible weight gain, oedema, and problems in the cardiovascular and central nervous systems [4]. These symptoms are related to *α*-amylase inhibition, which leads to an increase in the abnormal bacterial fermentation of undigested carbohydrates in the colon [9]. Because of the negative side effects caused by some diabetic therapeutic drugs, there is an urgent need to find natural and safe alternatives derived from diet to inhibit *α*-amylase and *α*-glucosidase. Peptides derived from hydrolyzed proteins possess various physiological functions and have been reported to be effective inhibitors of carbohydrate-hydrolyzing enzymes [1,10], with additional antioxidant, antimicrobial, antihypertensive, and antiproliferative properties [11]. Protein peptides with this biological activity are mostly released through the enzymatic hydrolysis process [12]. The bioactive peptides perform their functions through a specific amino acid sequence ranging from 2 to 50 amino acid residues [13].

Peanut (*Arachis hypogaea*) is a major agricultural crop that contains 47% to 55% high-value protein after oil extraction with a significant amino acid content, particularly essential amino acids [14]. The protein content of the product may increase depending on the precipitation method used and the purpose of production. Response surface methodology (RSM) is a multivariate statistic technique employed to improve a variety of processes, including food processing and enzymatic hydrolysis. RSM can avoid the limitations of traditional methods, which neglect the interactive effects between the parameters, and increase experiments cost and time [15]. To the best of our knowledge, no previous research on antidiabetic peptides of enzymatically hydrolyzed peanut protein has been conducted. Therefore, the current study aimed to optimize the enzymatic hydrolysis parameters of peanut protein and its antidiabetic properties using response surface methodology (RSM) with two proteases. In addition, protein hydrolysates prepared under optimal conditions were tested for their ability to inhibit the activity of *α*-amylase and *α*-glucosidase, as well as their antioxidant properties.

## 2. Materials and Methods

### 2.1. Materials and Reagents

The peanut protein powder, which contained 95% protein, was provided by Xi’an Dongchi Biotechnology Co., Ltd. (Xi’an, China). Alcalase from *Bacillus licheniformis* with the activity of 200 U/mg was obtained from Shanghai D&B Biological Science and Technology Co., Ltd. (Shanghai, China), and trypsin from porcine pancreas with activity of 250 U/mg was purchased from Shanghai Macklin Biochemical Co., Ltd. (Shanghai, China). Pre-stained protein with a molecular weight of 5 to 245 kDa was acquired from (M/S. Peqlab Ltd., Crableck Lane, Salisbury, UK). 1,1-Diphenyl-2-picrylhydrazyl (DPPH) radical and 2,2-azino-bis(3-ehtylbenzothiazoline-6-sulfonic acid) (ABTS) were purchased from Sigma Chemical Co. (Shanghai, China). *α*-Amylase and *α*-glucosidase were obtained from Coolaber Science and Technology Co., Ltd. All other reagents used were of the analytical grade.

### 2.2. Experimental Design for Optimization

The response surface methodology (RSM) was used in the current study by the Design Expert software V11.1.2.0 (Stat-Ease Inc., Minneapolis, MN, USA) with a central composite design (CCD), and the experimental design method was displayed in Table 1. Based on the preliminary results we obtained, four different independent parameters affecting the enzymatic hydrolysis were chosen as variables to be optimized, namely: Solid/liquid ratio (S/L, X_1_), enzyme/substrate ratio (E/S, X_2_), pH (X_3_), and reaction temperature (X_4_) at three levels (−1, 0 and +1) containing three replicates at the center point (Table 1). 

Degree of hydrolysis (DH; %, Y_1_), *α*-amylase inhibitory activity (%, Y_2_), and *α*-glucosidase inhibitory activity (%, Y_3_) were determined as the response variables that could be achieved from the enzymolysis of peanut protein.

The CCD with 30 patterns and three replicates of the central point was used. In order to examine the data, a quadratic polynomial regression model was used, as displayed:

(1) $$Y=\beta_{0}+\sum^{\;}\beta_{i}X_{i}+\sum^{\;}\beta_{ii}Xi^{2}\;\sum^{\;}\beta_{ij}X_{i\;}X_{j}$$

where *Y* is the response variable, *β_0_* is constant, *β_i_*, *β_ii_*, and *β_ij_* are the linear, quadratic, and interaction model coefficients, and *X_i_* and *X_j_*, respectively, are the independent variables. 

The enzymolysis process was carried out in a water bath equipped with a shaker to stir the reaction mixture. According to the required pH, the protein sample was mixed with sodium phosphate buffer or tris-HCl buffer (50 mM), and the enzyme was added when the mixture reached the specified temperature. Following the specified reaction time, the hydrolysate mixture was placed for 15 min in a water bath at 90 °C to prevent further enzymatic activity. The centrifugation step was completed in 15 min at 11,200× *g* and 4 °C (Avanti J-26S XP centrifuge, Beckman Coulter, Inc., Brea, CA, USA). The resulting supernatant was freeze-dried in a freeze dryer (LGJ-10 freeze-dryer, Guangzhou Feidi Biotechnology Co., Ltd., Guangdong, China) for 48 h at −60 °C with a vacuum of 0.12 mbar. The lyophilized peanut protein hydrolysates were referred to alcalase hydrolysate (AH) and trypsin hydrolysate (TH), which were kept in airtight plastic bags at −20 °C until further investigation of the final products.

### 2.3. Response Parameters Analysis

#### 2.3.1. Degree of Hydrolysis Determination

The pH-stat method was used to determine the DH of peanut protein by the amount of sodium hydroxide (NaOH) used to keep the pH constant during the enzymolysis process. The DH values were calculated using Equation (2) defined by Adler-Nissen [16].

DH (%) = h/h_tot_ × 100 = (BN_b_/*α*h_tot_ M_p_) ×100 (2)

where h is the number of peptide bonds cleaved, h_tot_ is the total number of peptide bonds per gram of peanut protein (7.13 mM/g protein) [17], B is the volume of NaOH (mL) solution used, Nb is the normality of the NaOH (0.1N), MP is the protein mass (g) in the sample used, and *α* is the average dissociation degree of the terminal amino peptides, which was calculated by Equation (3):

*α* = (10^(pH-pk)^/1 + 10^(pH-pk)^) (3)

where pH and pK are the values at which the enzymolysis of protein was conducted.

#### 2.3.2. α-Amylase Inhibition Assay

The *α*-amylase inhibitory activity assay was executed by the iodine/potassium iodide (IKI) procedure described by Bahadori et al. [18] with some modifications. Briefly, 25 μL of sample solution was mixed with 50 μL of 0.5 mg/mL *α*-amylase solution dissolved in phosphate buffer (50 mM, pH 6.9) in 96-well microplates. The reaction was carried out after incubating the mixture for 10 min at 37 °C by adding starch (50 μL, 0.05%). The blank sample was made by mixing the sample with all of the other reagents except the *α*-amylase enzyme solution and incubating the mixture at 37 °C for 20 min. To stop the reaction, 25 μL of HCl (1M) was used, and then 100 μL of IKI solution was added. The absorbance of the sample and blank was read at 630 nm using Infinite M200 PRO (Tecan Austria GmbH, Grödig, Austria), and the *α*-amylase inhibitory was calculated as the following:

(4) $$\alpha -\mathrm{amylase}\;\mathrm{inhibitory}\;\mathrm{activity}\;\left(\%\right)=\frac{A_{B}-A_{S}}{A_{S}}\times 100$$

where: *A_B_* and *A_S_* are the absorbance of reaction blank and sample.

The IC_50_ value, which is defined as a concentration of protein hydrolysate sufficient to inhibit 50% of the *α*-amylase activity, has been calculated according to the activity of different concentrations using Excel 2016 (Microsoft, Redmond, WA, USA).

#### 2.3.3. α-Glucosidase Inhibition Assay

The assay of *α*-glucosidase inhibitory was carried out according to the method of Ramadhan et al. [11] with some modifications. Carefully, 20 μL of the sample was mixed with 10 μL of *α*-glucosidase solution (1 U/mL, 50 mM phosphate buffer pH 6.9). This mixture was incubated at 37 °C for 20 min, and then 10 μL of *p*-nitro phenyl glucopyranoside (pNPG, 5 mM) dissolved in 50 mM phosphate buffer pH 6.9 was added to the mixture as a substrate to start the reaction and incubated for 20 min at 37 °C. Subsequently, 75 μL of Na_2_CO_3_ solution (1.0 M) was added to inhibit the reaction. The blank sample was prepared by mixing the same materials with replacing the hydrolysate sample with a buffer solution. The absorbance of the released product (*p*-nitro phenol) was measured at 405 nm using Infinite M200 PRO (Tecan Austria GmbH, Grödig, Austria) to estimate the enzyme activity, and *α*-glucosidase inhibitory was determined according to the following formula:

(5) $$\alpha -\mathrm{glucosidase}\;\mathrm{inhibitory}\;\mathrm{activity}\;\left(\%\right)=\frac{A_{B}-A_{S}}{A_{B}}\times 100$$

where: *A_B_* and *A_S_* are the absorbance of the blank sample and sample.

The IC_50_ value of AH and TH against *α*-glucosidase activity was calculated.

### 2.4. Protein Patterns by SDS-PAGE

For molecular mass distribution of peanut protein and its hydrolysates (AH and TH), Sodium dodecyl sulfate-polyacrylamide gel electrophoresis (SDS-PAGE) was done by following the method described by Laemmli [19] using electrophoresis (PowerPac Basic, Bio-Rad, Singapore). The sample solutions (20 μL) were loaded onto acrylamide stacking gel (4%), and acrylamide separating gel (15%) was used. Gels were run at a constant voltage for 5 min at 0.02 A, and then it was increased to 0.04 A for 1 h. The protein bands are highlighted by staining with Coomassie blue fast staining solution (Beyotime, Shanghai, China), then washing with water. Approximate molecular mass of lyophilized AH and TH was estimated by comparison with standards of molecular weight of 5–245 kDa purchased from Beijing kangrunchengye Biotechnology Co., Ltd. (Beijing, China).

### 2.5. Antioxidant Activity

#### 2.5.1. DPPH Radical-Scavenging Activity (DPPH-RSA)

The DPPH^•^ scavenging activity of AH and TH was estimated by a modified method described by Wang et al. [20] with some modifications. Different concentrations of AH and TH were initially prepared by dissolving the samples in deionized water (2, 4, 6, 8, and 10 mg/mL). The sample solutions (500 µL) and 500 µL of ethanol were mixed with the DPPH^•^ solution (125 µL, 0.2 mM in 95% methanol). The resulting mixtures were vortexed and incubated for 30 min in the absence of light at ambient temperature, and the absorbance of samples (*A_S_*), control sample (*A_C_*, DPPH solution without sample), and blank sample (*A_B_*, sample with methanol) was read at 517 nm. The following formula was used to calculate the DPPH’s scavenging activity:

(6) $$\mathrm{DPPH}\;\mathrm{radical}-\mathrm{scavenging}\;\mathrm{activity}\;\left(\%\right)=\frac{A_{C}+A_{B}-A_{S}\;}{A_{B}}\times 10$$

The IC_50,_ defined as the AH and TH concentration sufficient to scavenge DPPH^•^ was calculated

#### 2.5.2. ABTS Radical Cation Scavenging Activity (ABTS-RSA)

The ABTS^•+^ Scavenging activity of AH and TH was identified following the method of Liu et al. [21] with some modifications. A stock solution of ABTS (7 mM) and potassium persulfate (2.45 mM) was prepared (1:1 *v*/*v*) for 16 h at 25 °C without light exposure. With 98% ethanol, the absorbance of the mixed solution was adjusted to 0.70 ± 0.02 at 734 nm. Five concentrations of each hydrolysate (AH and TH) were prepared (2, 4, 6, 8, and 10 mg/mL), and then 5 µL of each concentration was mixed with 200 µL of ABTS^•+^ working solution. The resulting reaction solution was stood for 10 min in the dark. Then the absorbance of the studied samples (*A_S_*) and control sample (*A_C_*, deionized water was used in place of the protein hydrolysate solution) was estimated at 734 nm, and ABTS^•+^ scavenging activity of AH and TH samples was determined using the next equation.

(7) $$\mathrm{ABTS}\;\mathrm{radical}\;\mathrm{scavenging}\;\mathrm{activity}\;\left(\%\right)=\frac{A_{C}-A_{S}}{A_{C}}\times 100$$

The IC_50,_ defined as the AH and TH concentration sufficient to scavenge ABTS^•+^ was calculated.

### 2.6. Data Analysis

All assays were performed in triplicates and the experimental results were expressed as means ± SD. The data were fitted to a second-order polynomial regression model with linear, quadratic, and interaction terms. An analysis of variance (ANOVA) was performed with a 95% confidence level for each response variable to determine the significance and suitability of the model. Statistical significance was determined by computing the *F*-value using probability (*p*) of 0.05 for all polynomial terms.

The actual values were compared with the predicted values by calculating the residual standard error (RSE), according to Equation (8), in which RSE values are considered significant when their values less than 0.05.

(8) $$\mathrm{RSE}\;\left(\%\right)=\frac{\mathrm{Actual}\;\mathrm{value}-\mathrm{Predicted}\;\mathrm{value}}{\mathrm{Predicted}\;\mathrm{value}}\times 100$$

The difference between AH and TH of the optimized products was statistically analyzed using a *t*-test between different concentrations of antioxidant activities and antidiabetic.

## 3. Results and Discussion

### 3.1. Optimization of Enzymolysis Conditions

**Figure 1:**
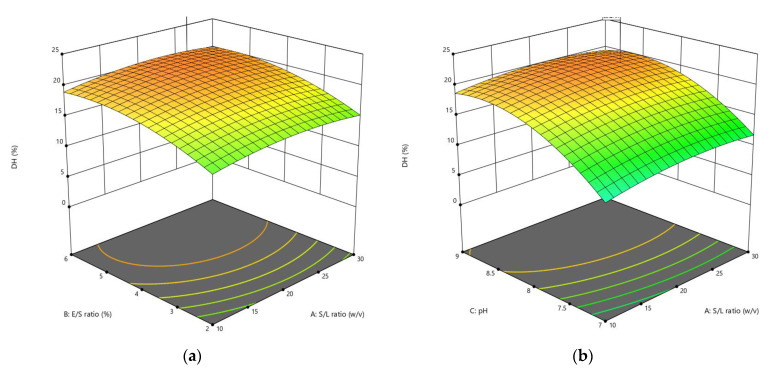

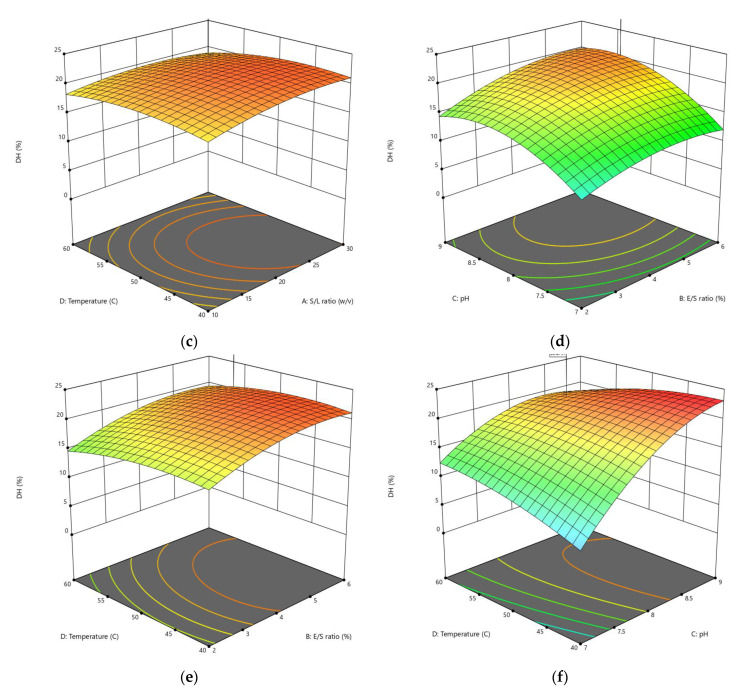
Three-dimensional (3D) response surface plots showing the influence of variable parameters on DH response using alcalase: (**a**) E/S ratio vs. S/L ratio, (**b**) pH vs. S/L ratio, (**c**) temperature vs. S/L ratio, (**d**) pH vs. E/S ratio, (**e**) temperature vs. S/L ratio and (**f**) temperature vs. pH.

**Figure 2:**
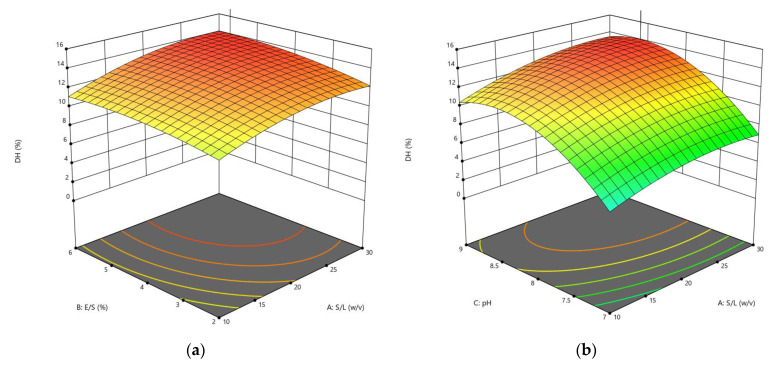

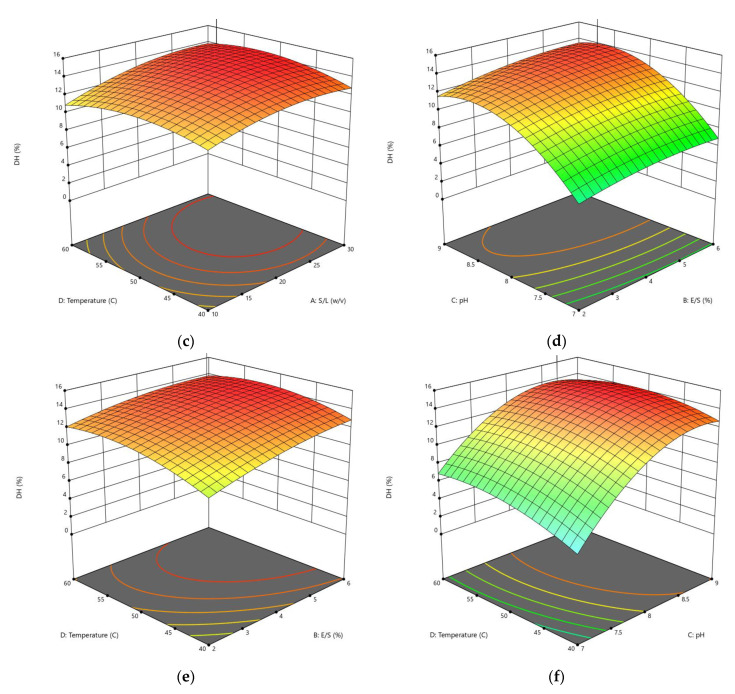
3D response surface plots showing the influence of variable parameters on DH response using trypsin: (**a**) E/S ratio vs. S/L ratio, (**b**) pH vs. S/L ratio, (**c**) temperature vs. S/L ratio, (**d**) pH vs. E/S ratio, (**e**) temperature vs. S/L ratio and (**f**) temperature vs. pH.

**Figure 3:**
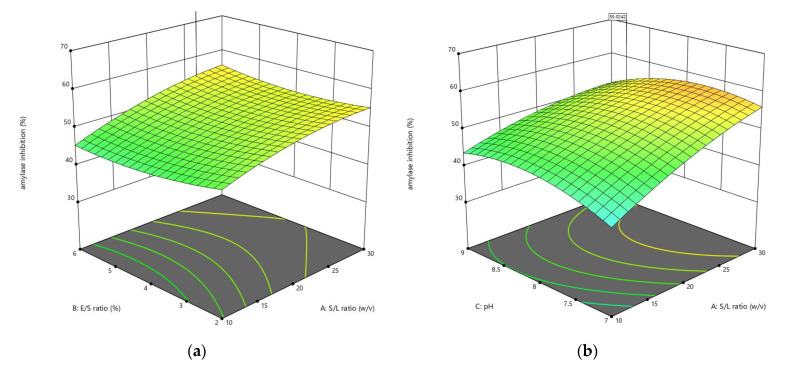

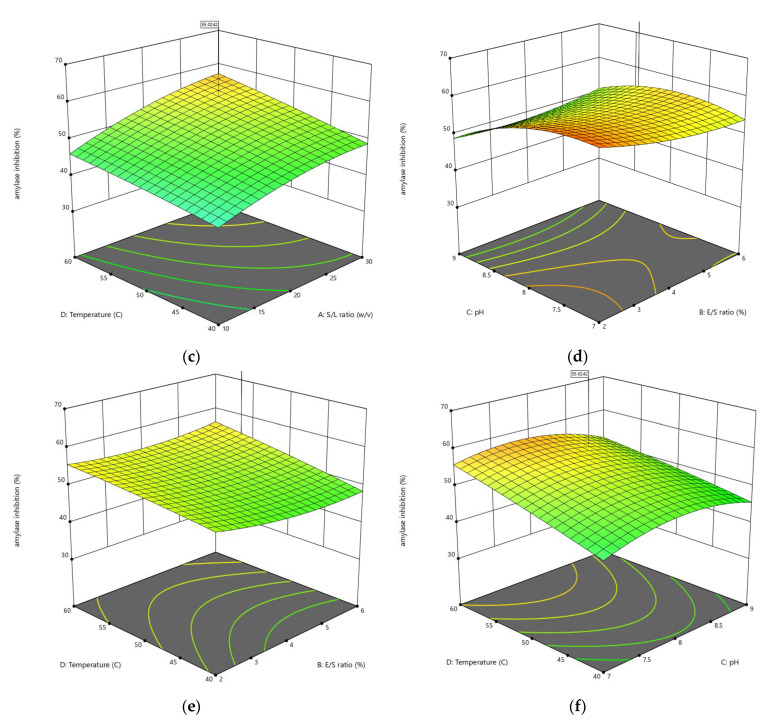
3D response surface plots showing the influence of variable parameters on the *α*-amylase inhibition activity response using alcalase: (**a**) E/S ratio vs. S/L ratio, (**b**) pH vs. S/L ratio, (**c**) temperature vs. S/L ratio, (**d**) pH vs. E/S ratio, (**e**) temperature vs. S/L ratio and (**f**) temperature vs. pH.

**Figure 4:**
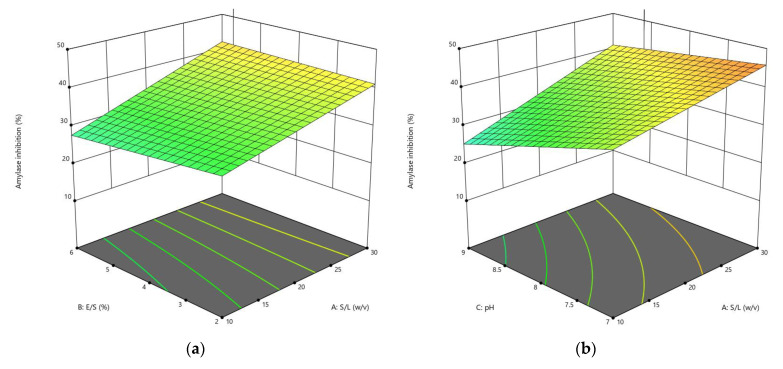

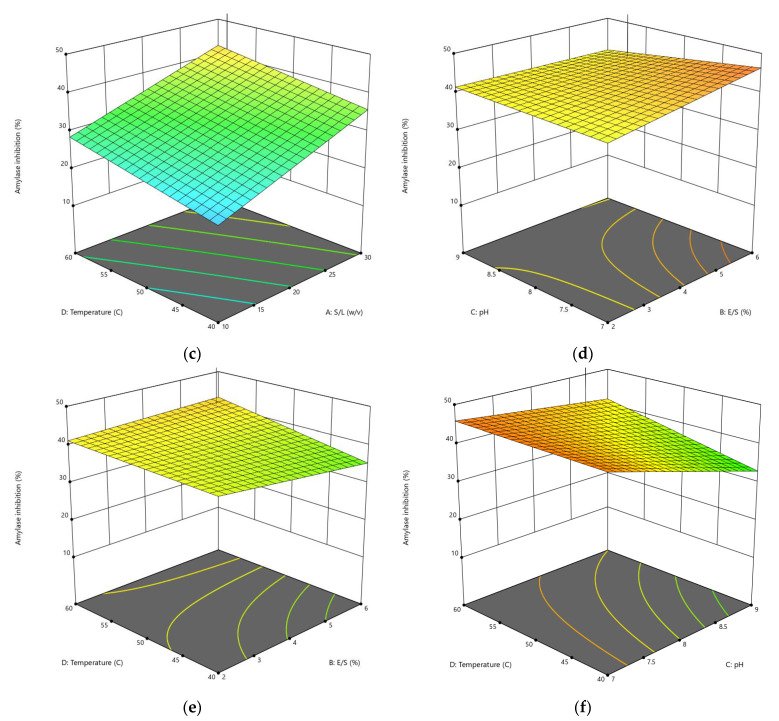
3D response surface plots showing the influence of variable parameters on the *α*-amylase inhibition activity response using trypsin: (**a**) E/S ratio vs. S/L ratio, (**b**) pH vs. S/L ratio, (**c**) temperature vs. S/L ratio, (**d**) pH vs. E/S ratio, (**e**) temperature vs. S/L ratio, and (**f**) temperature vs. pH.

**Figure 5:**
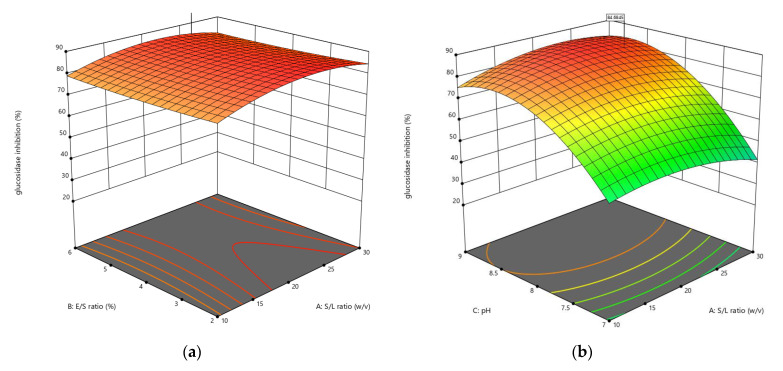

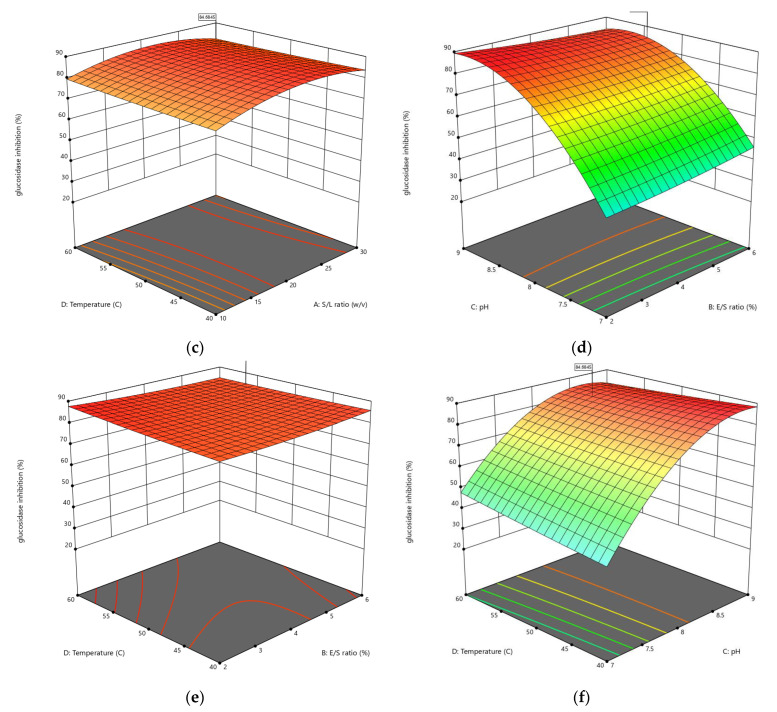
3D response surface plots showing the influence of variable parameters on the *α*-glucosidase inhibition activity response using alcalase: (**a**) E/S ratio vs. S/L ratio, (**b**) pH vs. S/L ratio, (**c**) temperature vs. S/L ratio, (**d**) pH vs. E/S ratio, (**e**) temperature vs. S/L ratio and (**f**) temperature vs. pH.

**Figure 6:**
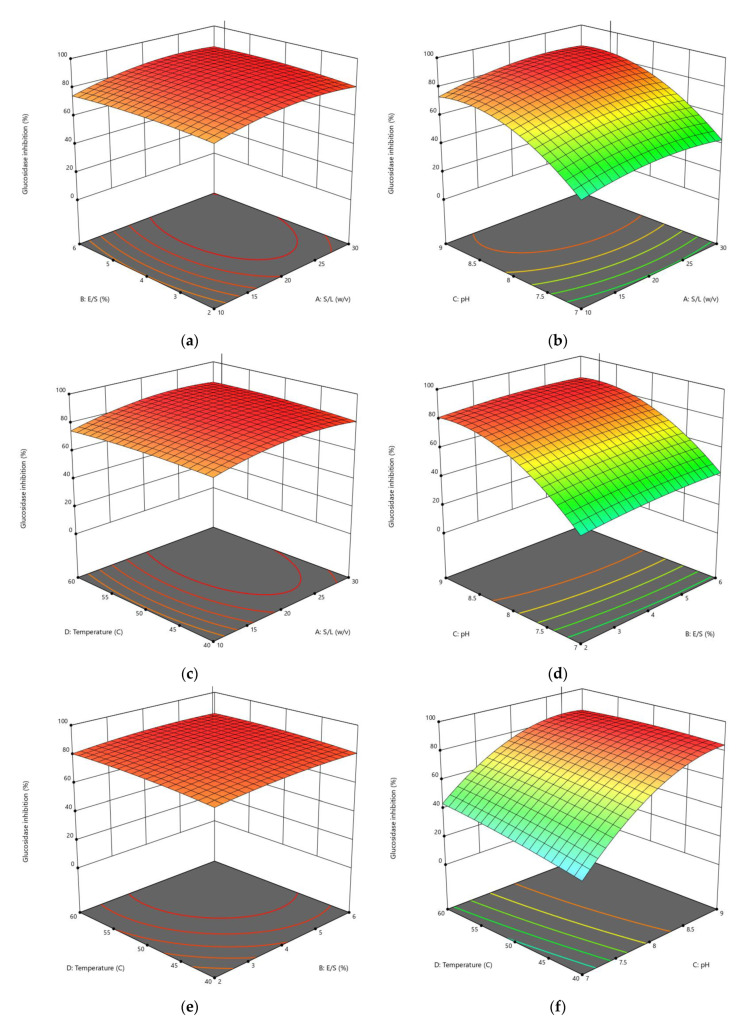
3D response surface plots showing the influence of variable parameters on the *α*-glucosidase inhibition activity response using trypsin: (**a**) E/S ratio vs. S/L ratio, (**b**) pH vs. S/L ratio, (**c**) temperature vs. S/L ratio, (**d**) pH vs. E/S ratio, (**e**) temperature vs. S/L ratio and (**f**) temperature vs. pH.

The CCD was applied to optimize the enzymolysis of peanut protein using two proteases (alcalase and trypsin) by combining four different independent variables (S/L, E/S, pH, and reaction temperature). The DH, *α*-amylase inhibition, and *α*-glucosidase inhibition were estimated using 30 different combinations of these variables. RSM was used to generate regression Equations (1)–(6), which explain the major and interactive influences of independent variables in coded units on response variables. Table 2 displays the statistical analysis of the model equations, as well as the significance of each coefficient identified by the *F*-test and *P*-value. As illustrated in Figure 1, Figure 2, Figure 3, Figure 4, Figure 5 and Figure 6, four independent factors were used in our study for simultaneous analysis of their effects on DH and antidiabetic activities (*α*-amylase inhibition activity and *α*-glucosidase inhibition activity).

#### 3.1.1. Influences of Parameters on the DH

The proteolysis was determined as the DH, which refers to the percentage of peptide bonds cleaved by the enzyme used. In the current study, the model was significant (*p* ˂ 0.0001) and thus could be used to optimize the hydrolysis process using alcalase and trypsin, and the coefficient of determination (*R*^2^) was 0.9127 and 0.9623, respectively, indicating good agreement between the actual and predicted values of DH. The parameters of the response surface model included in Equations (9) and (10) for alcalase and trypsin, respectively, were evaluated, and the statistical analysis revealed that all four hydrolysis factors (S/L, E/S, pH, and temperature) had a significant impact on the % DH using trypsin, while the E/S ratio and pH had a significant influence using alcalase.

Y_1_ = 19.229 + 0.866X_1_ + 1.996X_2_ + 4.960X_3_ + 0.448X_4_ + 0.073X_1_X_2_ − 0.258X_1_X_3_ − 0.478X_1_X_4_ + 0.696X_2_X_3_ + 0.049X_2_X_4_ − 2.571X_3_X_4_ − 1.019X_1_^2^ − 1.723X_2_^2^ − 3.403X_3_^2^ − 1.134X_4_^2^ (9)

Y_1_ = 12.765 + 0.837X_1_ + 0.903X_2_ + 3.326X_3_ + 0.752X_4_ + 0.173X_1_X_2_ + 0.238X_1_X_3_ + 0.350X_1_X_4_ +0.185X_2_X_3_ − 0.337X_2_X_4_ − 0.543X_3_X_4_ − 0.907X_1_^2^ − 0.529X_2_^2^ − 2.840X_3_^2^ + 1.113X_4_^2^ (10)

The significance model also can be described through the *F*-value and P-value. The F values were 11.20 and 27.31, respectively, while the *P* values were less than 0.0001 in both hydrolysates, thus, these values indicate the significance model (Table 2). Bahari et al. [22] reported that a high F-value with a low P-value indicates that the independent variables have a significant effect on their respective responses. Figure 1 and Figure 2 show the 3D surface plots of the DH response as affected by each enzymolysis condition.

The enzymolysis conditions synergistically affect the reaction rate, and this may be attributed to their influence on the enzyme activity and enzymatic hydrolysis mechanism [23]. As a result, the actual levels of DH produced by the enzymatic digestion of peanut protein varied, ranging from 3.71% to 22.93% in AH and 0.86% to 14.38% in TH (Table 3). Figure 1a and Figure 2a correlate the interaction effect between the S/L ratio and E/S ratio at constant pH (8.89 and 8.56) and temperature (40.36 °C and 58.75 °C) for AH and TH, respectively. According to the results of the DH, it is clear that increasing the E/S ratio from 2% to 6% has a greater impact when using alcalase, whereas increasing these two factors on DH had a similar effect when using trypsin. See et al. [24] reported that increasing the E/S ratio had a greater impact on DH, therefore reducing hydrolysis time. In the interaction effects of pH and S/L ratio at constant E/S ratio (5.34% and 6.67%) and temperature (40.36 °C and 58.75 °C), the effect of increasing pH to the optimal point was much higher in both hydrolyses (Figure 1b and Figure 2b). 

The optimal pH depends on the substrate and E/S ratio used in hydrolysis. Thus, at certain levels of pH, denaturation of the enzyme protein structure may occur or an alteration in the ionic feature of the substrate used in the hydrolysis, both of which decrease the substrate’s capability to bind to the enzymes in the reaction mixture [12]. Figure 1c and Figure 2c represent the combined effects between temperature and S/L ratio at a constant E/S ratio (5.34% and 5.67%) and pH (8.89 and 8.56), respectively. The maximum DH was obtained at a temperature (40 °C and 50 °C) and an S/L ratio (1:30 and 1:20 *w*/*v*), respectively.

Increasing the temperature above the optimum point for enzyme activity decreases the DH, and this may be attributed to thermal denaturation of the protein, which reduces or inhibits enzyme activity to severe the peptide bonds [25]. The DH was significantly affected by the interaction of pH and E/S ratio, and the pH showed a higher effect in both hydrolysates as shown in Figure 1d and Figure 2d. The E/S ratio showed a higher effect on DH than the temperature in the AH (Figure 1e), while the effect of these two factors was close in the TH (Figure 2e). Last of all, Figure 1f and Figure 2f show the interaction between temperature and pH at a fixed S/L ratio (1:26.15 and 1:30 *w*/*v*) and an E/S ratio (5.34% and 5.67%). The highest DH can be achieved at a pH of around 8.5 for both hydrolysates. Alcalase showed a higher DH (22.93%) under optimum conditions during the optimization process than trypsin (14.38%). This variation in DH could be attributed to the specificity of the enzyme in cleaving the peptide bonds [26].

#### 3.1.2. Influences of Parameters on α-Amylase Inhibition

Figure 3 and Figure 4 show the response surface plots for the effects of hydrolysis conditions on the *α*-amylase inhibition activity by fixing two factors at their central levels and varying the other two factors in order to clarify their major and interactive effects on *α*-amylase inhibition.

Y_2_ = 51.491 + 4.631X_1_ − 2.099X_2_ − 1.387X_3_ + 2.649X_4_ + 0.906X_1_X_2_ − 2.193X_1_X_3_ + 0.985X_1_X_4_ + 1.999X_2_X_3_ + 1.036X_2_X_4_ − 1.059X_3_X_4_ − 1.795X_1_^2^ + 1.559X_2_^2^ − 3.656X_3_^2^ − 0.350X_4_^2^ (11)

Y_2_ = 36.0935 + 5.215X_1_ − 0.996X_2_ − 5.546X_3_ + 1.290X_4_ + 1.183X_1_X_2_ +1.846X_1_X_3_ − 0.231X_1_X_4_ − 1.671X_2_X_3_ + 1.471X_2_X_4_ + 1.977X_3_X_4_ (12)

The effect of four independent variables of the response surface model on *α*-amylase inhibition included in Equations (11) and (12) using alcalase and trypsin, respectively, was investigated, and the statistical analysis results revealed that these variables had a significant impact on the *α*-amylase inhibition in TH, which ranged from 14.50% to 49.64% (Table 3). While the effect of E/S ratio and pH were significant in the AH, and the rate of *α*-amylase inhibition under the different combinations ranged from 30.53% to 63.26%. The response surface plots showed that the S/L ratio had a greater influence on *α*-amylase inhibition than E/S ratio (Figure 3a and Figure 4a) and pH (Figure 3b and Figure 4b). Likewise, in the interaction between temperature and S/L ratio (Figure 3c and Figure 4c) at a fixed E/S ratio (6% and 5.67%) and pH (8.41 and 8.56), respectively, the *α*-amylase inhibition raised when the temperature was raised from 40 °C to 60 °C, but at a lower rate than in the case of increasing the S/L ratio from 1:10 to 1:30 *w*/*v*. When the pH and E/S ratio increased in the AH (Figure 3d), the *α*-amylase inhibition rate changed slightly. However, increasing these variables in the TH exhibited a linear influence on *α*-amylase inhibition (Figure 4d). In the interaction of temperature with the E/S ratio (Figure 3e and Figure 4e), and the interaction of temperature with the pH (Figure 3f and Figure 4f), it can be noted that the effect of increasing the temperature from 40 °C to 60 °C on the *α*-amylase inhibition rate was more than the effect of increasing the E/S ratio from 2% to 6%, and the pH from 7 to 9 for both hydrolysates. The interaction of bioactive peptides released during hydrolysis with the enzyme active site explains the action of α-amylase, resulting in less substrate-enzyme interaction. As a result, the substrate interaction surface is smaller, and there is less contact with the *α*-amylase’s multiple attack actions [27]. The mechanism of bioactive peptides inhibiting *α*-amylase activity, on the other hand, can be elucidated through peptide binding to the allosteric site (chloride ion and calcium site) in the enzyme structure.

The altered conformation becomes unstable, preventing α-amylase from transforming the substrate to the product or making the enzyme’s substrate binding site unsuitable for binding the substrate [28].

#### 3.1.3. Influences of Parameters on α-Glucosidase Inhibition

The effect of the enzymatic hydrolysis conditions on the antidiabetic activity of AH and TH was studied, and three-dimensional response surface plots (Figure 5 and Figure 6) depict the relationship between *α*-glucosidase inhibition of peanut hydrolysates and experimental variables. Four parameters of the response surface model included in Equations (13) and (14) for alcalase and trypsin, respectively, were investigated, and the results of statistical analysis indicated that the E/S ratio and pH had a significant influence using alcalase, while all parameters of hydrolysis using trypsin had a significant impact on *α*-glucosidase inhibition, with *R*^2^ values of 0.9745 and 0.9776, respectively.

Y_3_ = 78.206 + 1.339X_1_ + 1.262X_2_ + 21.753X_3_ + 2.719X_4_ − 0.534X_1_X_2_ + 3.253X_1_X_3_ − 1.025X_1_X_4_ − 2.766X_2_X_3_ − 1.293X_2_X_4_ − 3.379X_3_X_4_ − 5.220X_1_^2^ + 1.753X_2_^2^ − 14.172X_3_^2^ + 0.257X_4_^2^ (13)

Y_3_ = 77.024 + 2.298X_1_ + 1.585X_2_ + 22.981X_3_ + 3.212X_4_ + 0.826X_1_X_2_ + 1.516X_1_X_3_ + 0.789X_1_X_4_−0.738X_2_X_3_ − 0.315X_2_X_4_ − 3.937X_3_X_4_ − 5.070X_1_^2^ − 1.862X_2_^2^ − 13.531X_3_^2^ + 1.480X_4_^2^ (14)

The interaction between the different enzymolysis conditions clearly affected the antidiabetic activity using *α*-glycosidase, where the inhibition rate ranged from 21.97% to 87.73% with alcalase, and from 14.54% to 84.82% with trypsin (Table 3). A marked increase in the responses of *α*-glucosidase inhibition was observed when the S/L ratio was increased from 1:10 to 1:30 *w*/*v* during its interaction with the E/S ratio at constant pH and temperature in both cases (Figure 5a and Figure 6a).

*α*-Glucosidase inhibition increased when increasing pH from 7 to 9 and S/L ratio from 1:10 to 1:30 *w*/*v*, reaching an optimum at about 8.5 and 1:30 *w*/*v*, respectively, before declining (Figure 5b and Figure 6b). This result could be due to an increase in the DH caused by the interaction between these two conditions, which showed an increase in DH with increasing pH, as shown in Figure 1b and Figure 2b. As shown in Figure 5c and Figure 6c, the effect of interaction between temperature and S/L ratio on *α*-glucosidase inhibition was plotted at a fixed E/S ratio (5.40% and 5.67%) and pH (8.93 and 8.56). In both hydrolysates, the S/L ratio had a greater effect on *α*-glucosidase inhibition than temperature. The interaction of pH and E/S ratio led to the greatest inhibition of *α*-glucosidase at constant S/L ratios (1:24.17 and 1:30 *w*/*v*) and temperatures (40.04 °C and 58.75 °C), reaching 87.73% and 84.82% for AH and TH, respectively (Figure 5d and Figure 6d). The *α*-glucosidase inhibition increased slightly in the interaction between temperature and E/S ratio, increasing from 40 °C to 60 °C and from 2% to 6%, respectively, indicating that these two factors did not achieve a significant increase, as shown in Figure 5e and Figure 6e. Regarding the temperature-pH interaction, it was clear that increasing the pH from 7 to 9 had a greater effect than increasing the temperature at a constant S/L and E/S ratio, as displayed in Figure 5f and Figure 6f. In general, peanut protein hydrolysates inhibited *α*-glucosidase more effectively than *α*-amylase in most designs and under optimal conditions.

The difference in α-glucosidase inhibition activity by bioactive peptides resulting from the enzymatic hydrolysis process could be attributed to the existence of some amino acids in the peptide structure, which is related to the DH, which varies depending on the specificity of the enzyme used [10].

#### 3.1.4. Verification and Optimization

**Figure 7:**
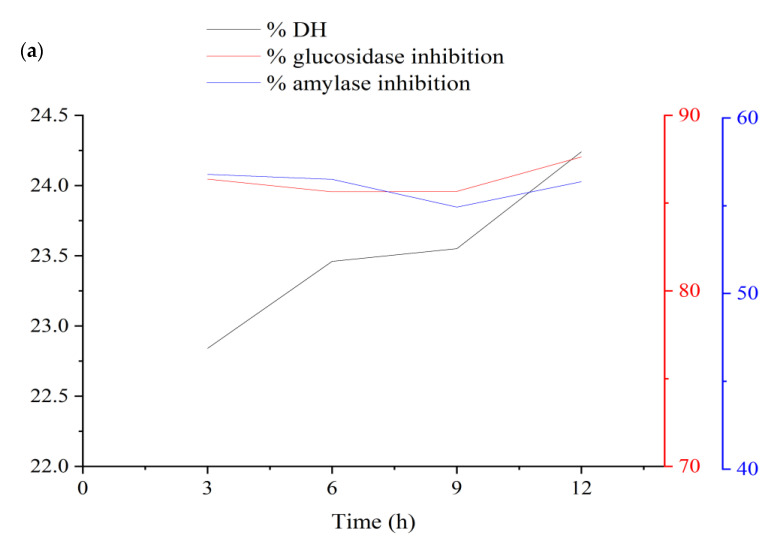

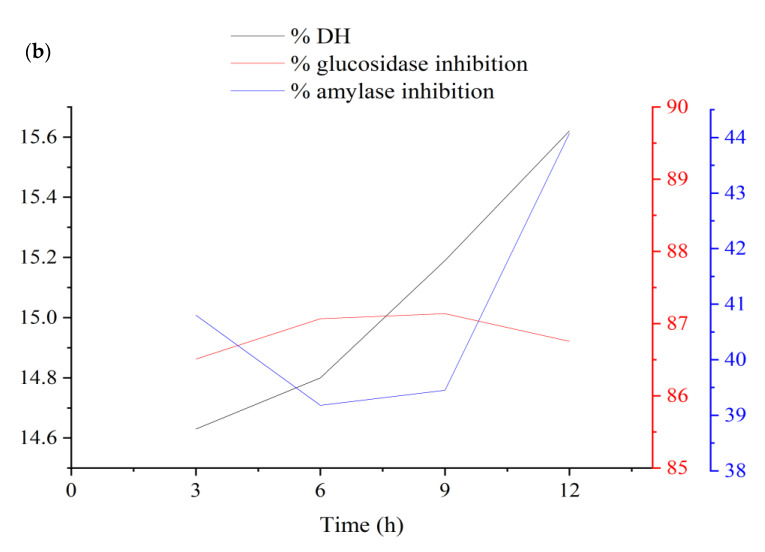
Effect of reaction time under optimal enzymolysis conditions on DH, *α*-amylase inhibition activity and *α*-glucosidase inhibition activity: (**a**) Using alcalase and (**b**) using trypsin.

After optimizing the four enzymatic hydrolysis conditions, the optimum hydrolysis time was tested, as displayed in Figure 7a for alcalase and Figure 7b for trypsin, and the results showed that three hours was the optimal time to achieve the optimal results for DH, *α*-amylase inhibition, and *α*-glucosidase as response variables combined. The best conditions for AH and TH were an S/L ratio of 1:26.22 and 1:30 *w*/*v*, an E/S ratio of 6% and 5.67%, a pH of 8.41 and 8.56, and a temperature of 56.18 °C and 58.75 °C, respectively. The experimental (actual) values of DH (22.84% and 14.63%), *α*-amylase inhibition (56.78% and 40.80%), and *α*-glucosidase inhibition (86.37% and 86.51%) were obtained under the optimal condition at 3 h.

The predicted values indicated above didn’t differ significantly from the predicted values for the DH (20.48% and 14.02%), *α*-amylase inhibition (55.02% and 42.15%), and *α*-glucosidase inhibition (84.68% and 84.54%), respectively, where RSE (%) is not high and didn’t exceed 5% in most values of both hydrolysates as shown in Table 4. 

The highest *α*-amylase inhibition was related to AH, which can be related to the existence of some amino acids (proline, glutamine, asparagine, glycine, leucine, and lysine) in the peptide structure [10], whereas the inhibition of *α*-glucosidase did not differ significantly despite the difference in the DH, which was much higher using alcalase. According to Famuwagun et al. [29], the peptides with a molecular weight ˂5 kDa were more active in inhibiting *α*-amylase than the peptides >5 kDa, which can be explained by the DH, which was higher when alcalase was used. This activity could be attributed to the ability of small peptides to bind more easily to the active site of the enzyme [30].

The activity (%) of AH and TH obtained in this study against *α*-amylase and *α*-glucosidase was higher than the results obtained by Fadimu et al. [31] from lupine protein hydrolysates using Protamex. According to Chandrasekaran and de Mejia [32], the inhibitory activity of *α*-glucosidase varies depending on the structure of the peptide, with IC_50_ ranging from 1.78 mg/mL to 8.74 mg/mL for the isolated peptide from germinated chickpea protein hydrolysates. As Yu et al. [33] found a strong *α*-glucosidase inhibitory peptide in the hydrolysate of egg albumin with an Arg residue at the C-terminal, it is possible that hydrolysis of peanut protein using trypsin leads to similar peptides with high *α*-glucosidase inhibition activity. On the other hand, the acarbose activity to inhibit *α*-amylase and *α*-glucosidase (IC_50_ = 0.26 mg/mL) was higher than AH (6.77 and 6.28 mg/mL) and TH (5.86 and 5.64 mg/mL), respectively. The difference between synthetic inhibitors and protein hydrolysates is that synthetic inhibitors are pure compounds, while protein hydrolysates are protein/peptide and non-protein ingredients mixtures [9]. These findings demonstrate that AH and TH generated from enzymolysis may be helpful in glycaemic management in subjects with T2DM due to their inhibitory activity against *α*-amylase and *α*-glucosidase.

### 3.2. Protein Patterns by SDS-PAGE

**Figure 8:**
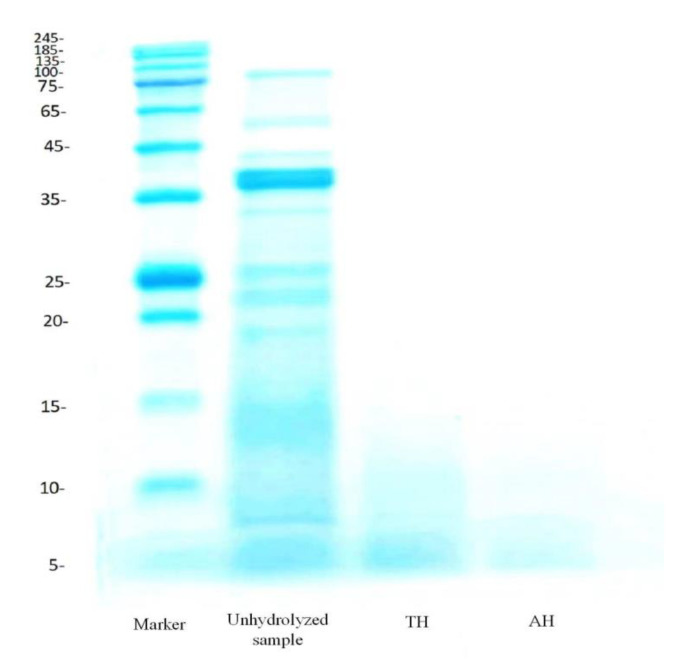
SDS-PAGE analysis on peanut protein and its hydrolysates using alcalase and trypsin.

Electrophoresis (SDS-PAGE) was used to obtain the molecular weight profiles of AH and TH and to confirm hydrolysis. The electrophoretic profiles demonstrated that the enzymolysis process was effective in the degradation of peanut protein, resulting in small peptides compared to the original protein, as shown in Figure 8. 

The major protein bands of original peanut protein ranged in molecular weight (MW) from 5 to 245 kDa. In contrast, TH’s MW was in the range of 15 kDa and was centered around the bond of 5 kDa, while the AH’s MW was less than 10 kDa. Enzymatic hydrolysis reduced the number of protein bands in both hydrolysates with increasing DH. Furthermore, as shown in Figure 8, the AH had a lower MW than the TH due to an increase in the DH using alcalase. Additionally, both hydrolysates with optimal DH lacked clear protein bands due to enzyme digestion of their molecules by alcalase and trypsin. Low molecular weights may be related to the degradation of peptide bonds, resulting in smaller peptides during the enzymolysis process [34]. This result is consistent with the findings of Phongthai et al. [35], who discovered that the bonds of rice bran protein hydrolysates disappeared when the DH was increased to 15%.

### 3.3. Antioxidant Activity of Hydrolysates

**Figure 9:**
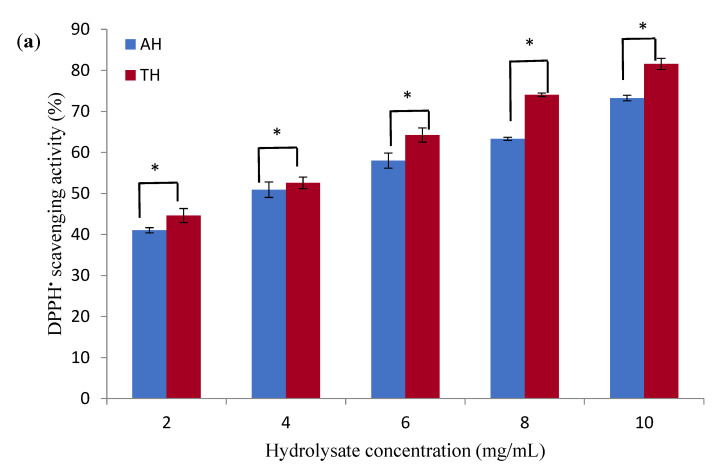

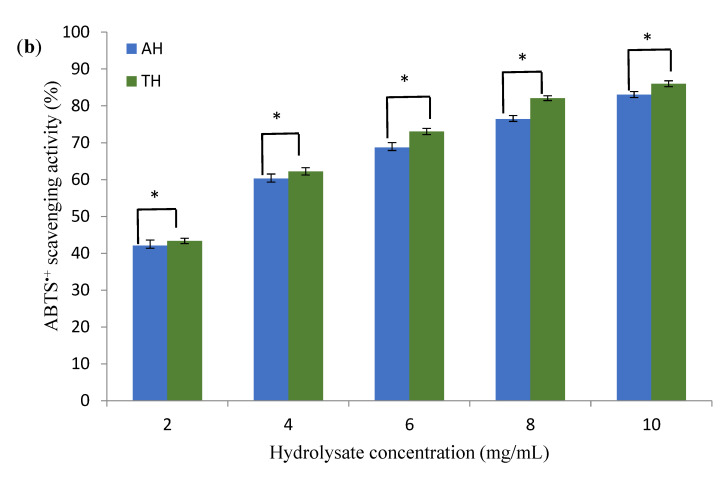
(**a**) DPPH^•^ scavenging activity and (**b**) ABTS^•+^ scavenging activity of peanut hydrolysates obtained under optimal conditions using alcalase (AH) and trypsin (TH). Data expressed as mean ± SD of triplicate determinations. (*) indicate significant differences at the same concentration with the different hydrolysates.

The antioxidant activities of optimized peanut protein hydrolysates (AH and TH) were investigated for DPPH-RSA and ABTS-RSA, and the results are given in Figure 9. Based on DPPH radical scavenging, a proton-donating component reduces DPPH solution absorbance at 517 nm by generating a diamagnetic molecule by receiving electrons from an antioxidant molecule. It was shown that the antiradical activity of AH and TH against DPPH^•^ increased significantly (*p* < 0.05) from 41.03% and 44.06% to 73.24% and 81.56% as hydrolysate concentration increased from 2 to 10 mg/mL. The antiradical activity of AH and TH against ABTS^•+^, on the other hand, ranged from 42.02% and 43.35% at 2 mg/mL to 83.06% and 86% at 10 mg/mL, respectively. Our findings corroborate previous research that reported an increase in antiradical activity as the concentration of protein hydrolysate increased [36,37]. TH was more active against DPPH and ABTS radicals (IC_50_ of 3.20 and 2.32 mg/mL) than AH (IC_50_ of 4.10 and 2.71 mg/mL), respectively. The activity of AH and TH against DPPH^•^ and ABTS^•+^ was higher than that found by Zaky et al. [34] from rice bran protein hydrolysate using flavourzyme, which was 17.2% and 35.1%, sequentially, but less than the chickpea hydrolysate activity (>70%) against DPPH^•^ [36], and mung bean protein hydrolysate (99.10%) against ABTS^•+^ [21] at 5 mg/mL. However, the samples of this study were more active than chicken skin gelatin hydrolysate (IC_50_ of 16.68 mg/mL) obtained using different enzymes [38]. Latorres et al. [39] stated that the ability of protein hydrolysate to scavenge free radicals is influenced by peptide size released during the enzymolysis process and the enzyme’s specificity for breaking peptide bonds.

## 4. Conclusions

In the current study, the CCD method and response surface methodology were applied to optimize the enzymolysis conditions of peanut protein for the best DH and antidiabetic activity. An analysis of variance revealed that a quadratic model significantly contributed to the response variables. Optimization results were obtained, and mathematical models were used to generate 3D response surfaces. The best conditions for preparing the optimized peanut protein hydrolysates using alcalase and trypsin were found to be a S/L ratio of 1: 26.22 and 1:30 *w*/*v*, an E/S ratio of 6% and 5.67%, a pH of 8.41 and 8.56, and a temperature of 56.18 °C and 58.75 °C, respectively. The maximum DH (22.84% and 14.63%), *α*-amylase inhibition (56.78% and 40.80%), and *α*-glucosidase inhibition (86.37% and 86.51%) were obtained at the optimum hydrolysis time (3 h), which were consistent with the predicted results. According to the current findings, AH and TH provided good DPPH^•^ and ABTS^•+^ scavenging activities, which could be due to the low molecular weight of cleaved peptides. This study provided valuable findings for optimizing AH and TH production, which could be used as natural antidiabetic sources in food and as bioactive ingredients. Furthermore, additional research should be conducted to determine the peptides responsible for these activities, and the health benefits of AH and TH, using animal models and simulating digestion conditions.

## Figures and Tables

**Table 1:** Independent variables, their coded and actual levels used in RSM for optimizing enzymolysis conditions using alcalase and trypsin.

Parameters	Coded Level		
	−1	0	+1
Solid/liquid ratio (*w*/*v*, X_1_)	1:10	1:20	1:30
Enzyme/substrate ratio (*w*/*w*, X_2_)	2	4	6
pH (X_3_)	7	8	9
Temperature (X_4_)	40	50	60

**Table 2:** Analysis of variance for three responses with two proteases.

Source	Sum of Squares	Degree of Freedom	Mean of Square	*F*-Value	*p*-Value
Alcalase					
DH					
Model	985.28	14	70.38	11.20	<0.0001
Residual	94.22	15	6.28		
Pure error	0.1070	5	0.0214		
Lack of fit	94.11	10	9.41	439.77	<0.0001
Total	1079.50	29			
*R* ^2^	0.9127		Adj. *R*^2^	0.8313	
α-Amylase inhibition					
Model	1101.75	14	78.70	5.81	0.0008
Residual	203.05	15	13.54		
Pure error	11.92	5	2.38		
Lack of fit	191.12	10	19.11	8.02	0.0165
Total	1304.79	29			
*R* ^2^	0.8444		Adj. *R*^2^	0.6991	
α-Glucosidase inhibition					
Model	12933.34	14	923.81	41.00	<0.0001
Residual	338.01	15	22.53		
Pure error	3.98	5	0.7954		
Lack of fit	334.04	10	33.40	42.00	0.0003
Total	13271.36	29			
*R* ^2^	0.9745		Adj. *R*^2^	0.9508	
Trypsin					
DH					
Model	433.92	14	30.99	27.31	<0.0001
Residual	17.02	15	1.13		
Pure error	0.0000	5	0.0000		
Lack of fit	17.02	10	1.70		
Total	450.94	29			
*R* ^2^	0.9623		Adj. *R*^2^	0.9270	
α-Amylase inhibition					
Model	1229.94	10	122.99	9.19	<0.0001
Residual	254.33	19	13.39		
Pure error	0.0000	5	0.0000		
Lack of fit	254.33	14	18.17		
Total	1484.27	29			
*R* ^2^	0.8287		Adj. *R*^2^	0.7385	
α-Glucosidase inhibition					
Model	14264.56	14	1018.90	46.72	<0.0001
Residual	327.15	15	21.81		
Pure error	0.6474	5	0.1295		
Lack of fit	326.50	10	32.65	252.17	<0.0001
Total	14591.71	29			
*R* ^2^	0.9776		Adj. *R*^2^	0.9567	

**Table 3:** Actual levels of independent variables along with the obtained values for the response variables.

Run	Independent Variables				Alcalase			Trypsin		
	X_1_	X_2_	X_3_	X_4_	Y_1_	Y_2_	Y_3_	Y_1_	Y_2_	Y_3_
1	30	2	7	60	8.45	63.26	38.19	3.88	34.75	35.54
2	20	4	8	50	18.64	54.72	79.38	2.70	37.53	23.61
3	30	6	9	60	15.03	52.68	84.97	3.52	49.44	25.72
4	10	2	9	40	15.39	43.11	79.50	9.34	32.32	75.17
5	10	2	7	40	3.71	49.58	21.97	12.60	34.79	75.73
6	30	6	7	60	11.71	57.51	41.05	3.75	39.98	27.84
7	20	4	8	50	18.99	55.14	77.28	3.42	37.01	24.87
8	34.14	4	8	50	22.86	56.50	77.53	5.36	37.55	37.54
9	5.86	4	8	50	13.28	34.94	58.80	9.84	33.43	82.21
10	20	4	8	50	18.72	51.97	77.41	12.60	34.79	75.73
11	20	6.82	8	50	20.56	52.06	78.25	12.60	34.79	74.85
12	30	2	9	60	10.10	49.31	83.59	12.60	34.79	75.73
13	10	2	9	60	12.66	48.38	81.70	11.18	45.23	78.73
14	10	6	9	60	17.04	43.75	78.23	8.82	27.91	73.46
15	20	4	8	50	18.74	51.97	77.41	14.38	40.62	78.34
16	30	6	9	40	22.93	43.33	85.87	11.15	31.43	78.54
17	10	6	9	40	21.12	44.24	76.56	12.60	34.79	75.73
18	10	2	7	60	7.63	48.43	45.90	0.86	48.42	14.54
19	30	2	7	40	4.19	53.06	23.97	12.66	34.62	77.29
20	20	4	8	50	18.85	51.89	78.73	8.91	36.64	75.18
21	20	4	8	35.86	13.25	47.02	78.17	12.60	34.79	75.73
22	20	4	8	64.14	22.43	50.21	80.06	8.03	25.69	59.76
23	30	6	7	40	6.05	46.57	32.56	4.34	39.50	38.45
24	20	4	8	50	18.59	51.97	77.41	11.35	14.50	78.13
25	20	4	6.59	50	4.90	44.84	23.97	12.92	38.55	81.01
26	10	6	7	40	5.19	30.53	43.77	10.79	26.55	75.23
27	10	6	7	60	9.90	43.26	50.03	12.14	40.44	84.82
28	30	2	9	40	17.96	47.67	87.73	6.35	49.64	41.47
29	20	4	9.41	50	21.71	39.16	76.56	10.20	39.29	75.22
30	20	1.17	8	50	12.76	52.80	81.98	13.72	32.98	75.71

X_1_, S/L; X_2_, E/S; X_3_, pH; X_4_, temperature; Y_1_, degree of hydrolysis; Y_2_, *α*-amylase inhibitory activity; Y_3_, *α*-glucosidase inhibitory activity.

**Table 4:** Actual and predicted values observed during validation experiments under optimal conditions.

Parameters	Alcalase		Trypsin	
	Predicted	Actual	Predicted	Actual
DH	20.48	22.84	14.02	14.63
*α*-amylase inhibition	55.02	56.78	42.15	40.80
*α*-glucosidase inhibition	84.68	86.37	84.54	86.51

## Data Availability

Data is contained within the article.
